# Specific siRNA Targeting Receptor for Advanced Glycation End Products (RAGE) Decreases Proliferation in Human Breast Cancer Cell Lines

**DOI:** 10.3390/ijms14047959

**Published:** 2013-04-11

**Authors:** AL-Madhagi Radia, AL-Madhagi Yaser, Xiaoqian Ma, Juan Zhang, Cejun Yang, Qiong Dong, Pengfei Rong, Bin Ye, Sheng Liu, Wei Wang

**Affiliations:** 1 Cell Transplantation and Gene Therapy Institute, Department of Radiology, the Third Xiang Ya Hospital of Central South University, Changsha 410013, China; E-Mails: ralmadhagi@hotmail.com (A.-M.R.); mxq8933@yahoo.com.cn (X.M.); gxl198501@163.com (J.Z.); yangcejun5358@yeah.net (C.Y.); dongqiong57@126.com (Q.D.); rongpengfei66@yahoo.com.cn (P.R.); yebin9992003@163.com (B.Y.); liushe0@hotmail.com (S.L.); 2 Center of Molecular Medicine, the First Xiang Ya Hospital of Central South University, Changsha 410078, China; E-Mail: yassnow@hotmail.com

**Keywords:** RAGE, siRNA, proliferation, breast cancer

## Abstract

Receptor for Advanced Glycation End Products (RAGE) is an oncogenic trans-membranous receptor overexpressed in various human cancers. However, the role of RAGE in breast cancer development and proliferation is still unclear. In this study, we demonstrated that RAGE expression levels are correlated to the degree of severity of breast cancer. Furthermore, there is a decrease in the proliferation of all sub-types of breast cancer, MCF-7, SK-Br-3 and MDA-MB-231, as a result of the effect of RAGE siRNA. RAGE siRNA arrested cells in the G1 phase and inhibited DNA synthesis (*p* < 0.05). Moreover, qRT-PCR and Western Blot results demonstrated that RAGE siRNA decreases the expression of transcriptional factor NF-κB p65 as well as the expression of cell proliferation markers PCNA and cyclinD1. RAGE and RAGE ligands can thus be considered as possible targets for breast cancer management and therapy.

## 1. Introduction

Breast cancer is the most common malignancy and the second leading cause of death in women [1,2]. The causes and pathogenesis of breast cancer are poorly understood [2]. Breast cancer may result from genetic alteration of normal cells [3–6]. However, some clinical and experimental data have suggested that chronic inflammation promotes mammary tumors [7].

Breast cancer is classified into several sub-types according to immunohistochemistry markers: estrogen receptor (ER) positive (luminal subtype), human epidermal growth factor receptor-2 (HER2) positive and triple-negative (basal-like) [2,8]. Triple-negative (basal like) sub-type, defined as tumors lacking estrogen (ER), progesterone (PR), and HER2 expression, accounts for 10%–17% of all breast carcinomas [9], shows remarkably high proliferation [10–12] and aggressive clinical behavior [13–15] and has a poor prognosis [2,16]. This sub-type relies on chemotherapy for treatment because it does not respond to hormonal or endocrine therapy, and unfortunately triple-negative breast cancer patients have worse outcomes after chemotherapy than patients with the other sub-types [17–19]. Carcinomas with HER2 overexpression have been reported in 18%–25% of human breast cancers [2,16] and show poor differentiation [2,20,21], and worse prognosis than luminal breast cancer [2]. ER positive (luminal sub-type) has been reported in 60%–70% of cases [2,8], is less aggressive, and has a better prognosis than the other two sub-types.

The Receptor for Advanced Glycation End products (RAGE) is a single transmembrane, multiligand receptor of the immunoglobulin family. It is usually expressed in low levels in a variety of cell types including immune cells, neurons, activated endothelial and vascular smooth muscle cells and bone forming cells [22–26], although high expression levels are found in the lungs and during embryonic development [27].

Some recent studies have reported a correlation between RAGE and different human pathologies including diabetes, neuronal degeneration, inflammation [13,28–30], Alzheimer’s disease, cardiovascular disease and cancers [24,31]. Other studies have linked the progression of many chronic diseases with RAGE-mediated proinflammatory processes including nephropathy [32,33], macrovascular disease, amyloidosis, inflammatory conditions (e.g., rheumatoid arthritis and inflammatory bowel disease) and sepsis [34,35].

RAGE has many ligands belonging to different family groups, such as the high mobility family group (including HMGB1) and some members of the S100 protein family group [23,36–38]. In primary breast cancer, high levels of HMGB1 were observed and enhanced by estrogen [39–41]. S100A4 has a direct correlation with mean vessel density in breast tumors [24,42] and S100P plays a role in the progression of breast cancer from initial tumorigenesis to invasive carcinoma [24]. The signal resulting from the interaction between RAGE and its ligands plays a role in the modulation of cancer cell functions by increasing tumor invasion and metastasis [43]. Furthermore, several studies have reported a strong correlation between RAGE expression and the malignant potentials of gastric cancer [44], colon cancer [45,46], common bile duct cancer [47], pancreatic cancer [48], and prostate cancer [49].

NF-κB (nuclear factor kappa-light-chain-enhancer of activated B cells) is a protein complex that controls DNA transcription. The NF-κB response arises from the binding of Rel/NF-κB complexes to exact DNA regulatory sites (κB sites) of target genes in various cell types [23,50,51]. Interactions between RAGE and its ligand stimulate signaling pathways (e.g., p38 and p44/42 MAP kinase [52]. NF-κB and cdc42/rac [53]). Incorrect regulation of NF-κB has been linked to cancer, inflammatory and autoimmune diseases, septic shock, viral infection, and improper immune development [54–57]. NF-κB stimulates proliferation in different cell types, including human breast cancers [58].

In this study we investigated the oncogenic role of RAGE and its correlation to proliferation in breast cancer cells. Three different breast cancer cell lines, MD-MBA-231 (triple-negative), SK-Br-3 (HER2 overexpression) and MCF-7 [estrogen receptor (ER) positive], were selected and studied for their expression of RAGE. RAGE siRNA was used to knockdown RAGE gene expression in these cell lines, and cancer cell proliferation was investigated and compared to a control.

## 2. Results and Discussion

### 2.1. RAGE Expression in Different Sub-Types of Breast Cancer Cell Lines

**Figure 1:**
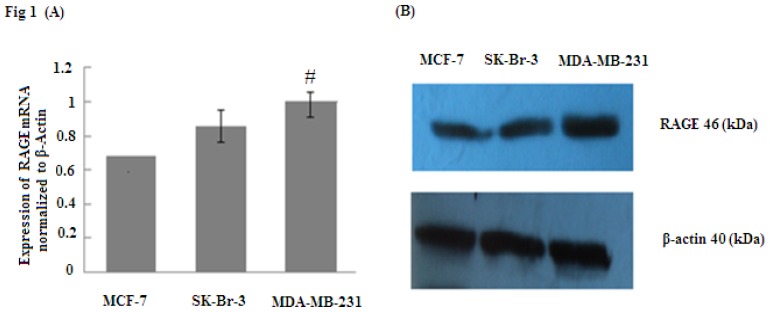
RAGE mRNA and protein expression (normalized to β-actin expression) in MCF-7, SK-Br-3, MDA-MB-231 cell lines analyzed by (**A**) qRT-PCR and (**B**) Western Blot. The results indicate that RAGE expression in MDA-MB-231 was significantly higher than in MCF-7 and SK-Br-3; (# *p* < 0.05). Three independent measurements were performed and averaged.

RAGE plays an essential role in the modulation of cancer cell functions when it is overexpressed through increasing tumor invasion and metastasis. To determine whether RAGE is overexpressed in breast cancer, RAGE mRNA was evaluated by quantitative real time PCR (qRT-PCR) and RAGE protein was detected by Western Blot. The results indicated that MCF-7, SK-Br-3 and MDA-MB-231 cell lines expressed RAGE mRNA and protein; however, MD-MBA-231(aggressive sub-type) expressed significantly higher levels of RAGE than SK-Br-3 and MCF-7 (# *p* < 0.05) (Figure 1). Thus, our results suggest that RAGE overexpression is closely associated with breast cancer.

### 2.2. Efficiency of RAGE Gene Knockdown

**Figure 2:**
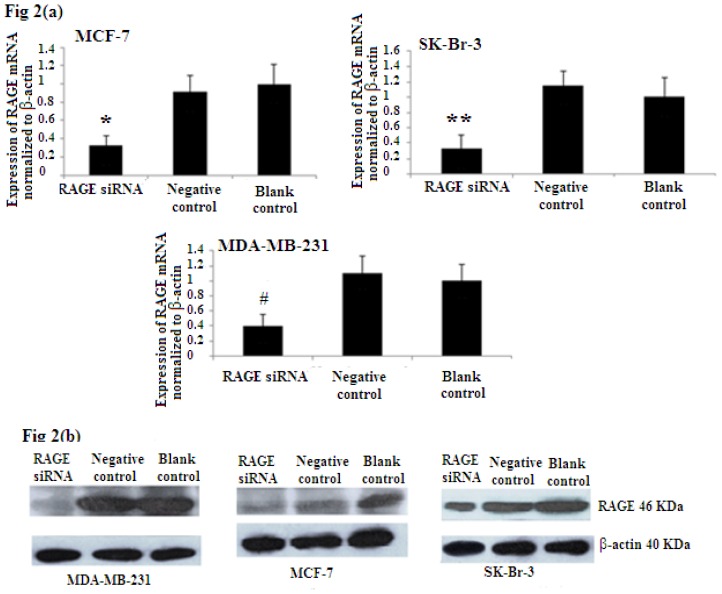
RAGE siRNA silencing efficiency by (**a**) qRT-PCR (*, **, # *p* < 0.05) and (**b**) Western Blot (*p* < 0.05). The results showed decreased expression of RAGE mRNA and protein in the siRNA group compared to the negative control and blank control groups. Three independent measurements were performed and averaged.

To evaluate the efficiency of RAGE siRNA in breast cancer sub-types, MCF-7, SK-Br-3, and MDA-MB-231 cells were transfected with siRNA or negative control RNA. qRT-PCR (Figure 2a) and Western Blot (Figure 2b) showed that RAGE siRNA significantly decrease the expression of RAGE mRNA and protein in all selected cell lines compared to the negative control and blank control.

### 2.3. RAGE siRNA Decreases Viability in Breast Cancer

**Figure 3:**
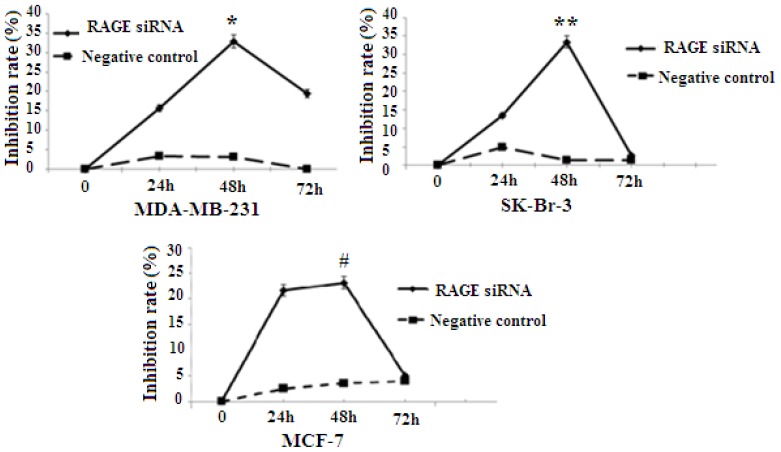
MTT [3-(4,5-dimethylthiazol-2-yl)-2,5-diphenyltetrazolium bromide] colorimetric result. RAGE siRNA inhibits proliferation in all cell lines (*, **, # *p* < 0.05). The highest growth inhibition is achieved after 48 h of incubation with RAGE siRNA. Three independent measurements were performed and averaged.

RAGE expression was found to be correlated with the proliferation of several types of cancer. To explore whether RAGE contributed to breast cancer cellular proliferation, MCF-7, SK-Br-3, and MDA-MB-231 were transfected with RAGE siRNA or negative control RNA, and cell proliferation was evaluated using the 3-(4, 5-dimethylthiazol-2-yl)-2, 5-diphenyltetrazolium bromide (MTT) assay. The results indicated that MCF-7, SK-Br-3 and MDA-MB-231 cells transfected with RAGE siRNA have a slower growth rate than cells transfected with negative control RNA (NC) or blank controls. In all cell lines the highest growth inhibition was achieved after 48 h of incubation with RAGE siRNA (Figure 3).

### 2.4. RAGE siRNA Induces G1 Arrest in Breast Cancer Cell Lines

**Figure 4:**
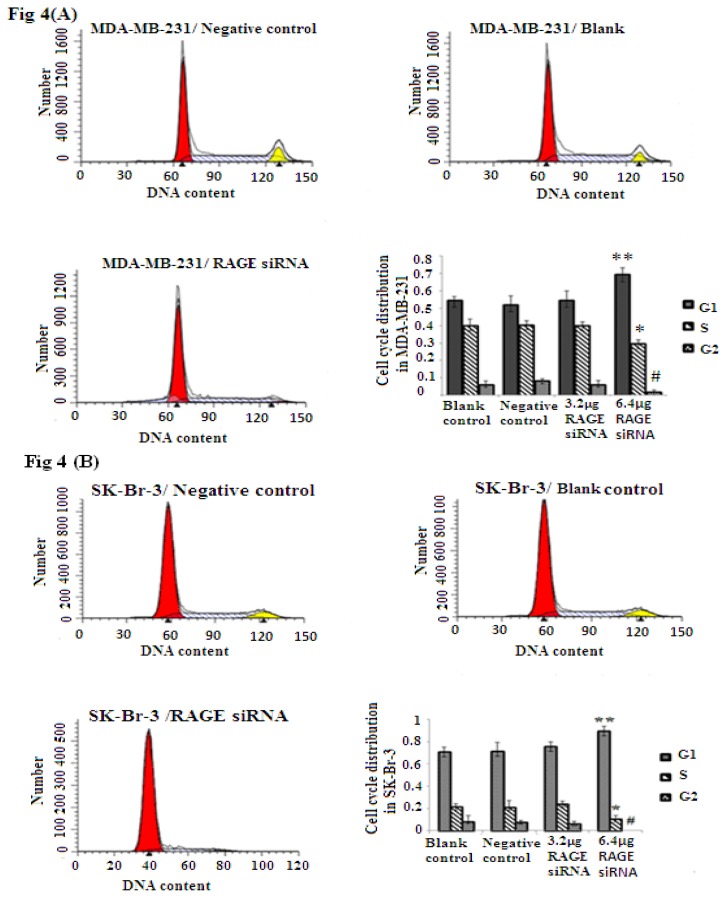

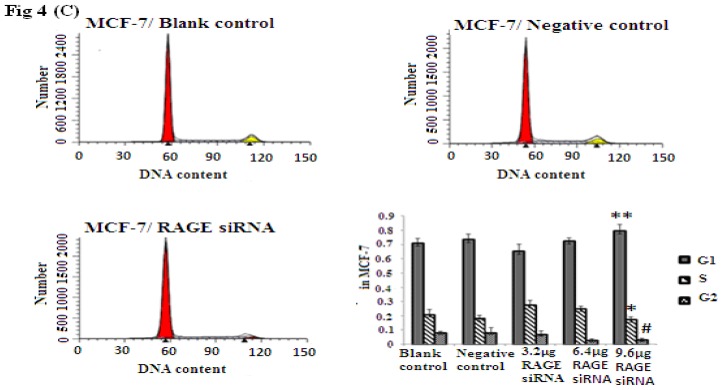
FACS flow cytometry staining studies in MCF-7, SK-Br-3 and MDA-MB-231 cell lines after treatment with RAGE siRNA. RAGE siRNA inhibited DNA synthesis, significantly increased the percentage of cells in G1 phase and significantly decreased the percentage of cells in the S and G2 phases 48 h post-treatment. In MDA-MB-231 (*, **, # *p* < 0.05) and SK-Br-3 (*, **, # *p* < 0.05) 3.2–6.4 μg of siRNA were needed to obtain significant results; however, MCF-7 (*, **, # *p* < 0.05) required 9.6 μg of siRNA to achieve a significant result. Three independent measurements were performed and averaged.

Cellular proliferation of breast cancer is closely related to RAGE expression as shown through the MTT assay results. To explore if RAGE expression has an influence on the cell cycle, we investigated the effect of RAGE siRNA on MCF-7, SK-BR-3 and MDA-MB-231 cells. The FACS results indicated that RAGE siRNA significantly increased the percentage of cells in G1 compared to negative control RNA (NC) or blank control. This increase in the G1 phase was coupled with a significant decrease in the percentage of cells in S and G2 after 48 h of transfection (*p* < 0.05). RAGE siRNA could arrest cells in the G1 phase at concentrations as low as 3.2–6.4 μg in MDA-MB-231(*, **, # *p* < 0.05) and SK-Br-3 (*, **, # *p* < 0.05); however, MCF-7 cells required a higher concentration (9.6 μg) of siRNA to show significant results (*, **, # *p* < 0.05, Figure 4).

### 2.5. Silencing RAGE Induces Negligible Apoptosis

**Figure 5:**
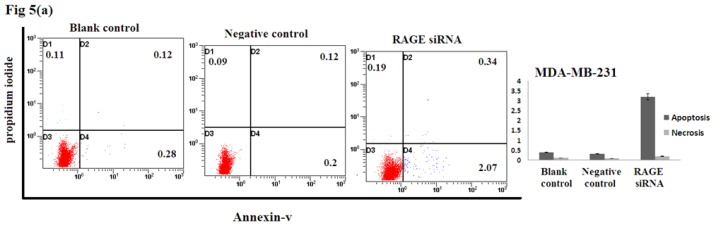

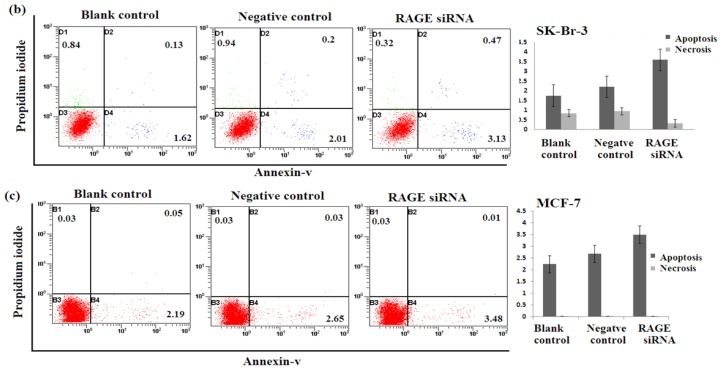
Effect of RAGE siRNA on apoptosis of (**a**) MDA-MB-231; (**b**) SK-Br-3; (**c**) MCF-7 breast cancers cell lines. Transfected cells were stained with Annexine-V and Propidium iodide followed by flow cytometry. Early apoptotic (right bottom), late apoptotic (right top) and necrotic (left top) cells were displayed. Three independent measurements were performed and averaged and no significant difference was detected.

Apoptosis assay of transfected breast cancer cell lines by RAGE siRNA investigated using Flow Cytometry. No significant difference of Annexin-V-positive apoptotic cells was detected in the RAGE siRNA-treated group compared to control groups as shown in Figure 5, suggesting that RAGE knockdown may not induce apoptosis of breast cancer cells.

### 2.6. RAGE siRNA Decreases Expression of PCNA and CyclinD1 mRNA

**Figure 6:**
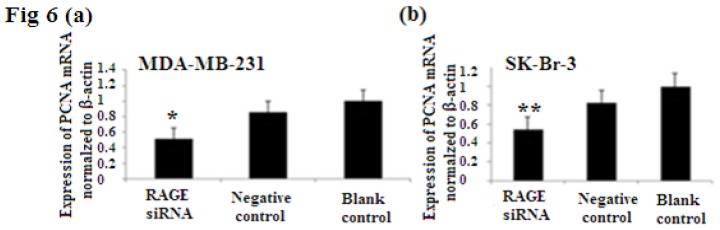

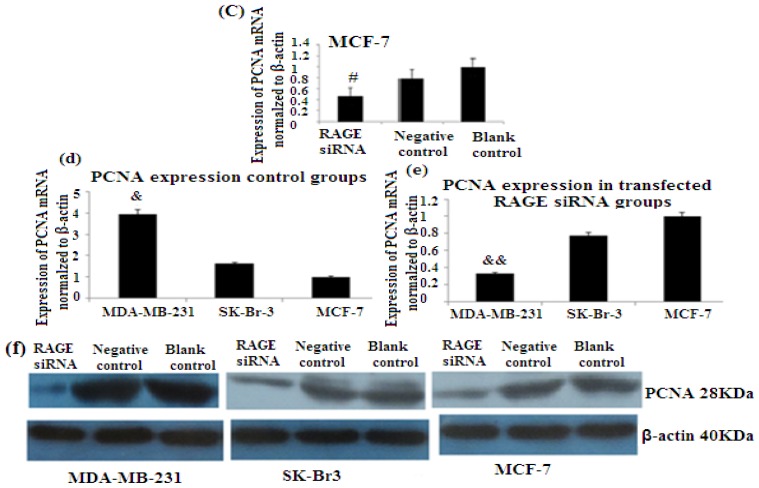
There was decreased expression of PCNA mRNA after transfection with RAGE siRNA as shown by qRT-PCR in MDA-MB-231 (**a**), SK-Br-3 (**b**) and MCF-7 (**c**) cell lines respectively (*, **, # *p* < 0.05) and Western Blot results (**f**) (*p* < 0.05) compared to the negative or blank control groups. Comparison between the expression of PCNA in the three cell lines in the control groups (**d**) and in the transfected groups (**e**). Three independent measurements were performed and averaged.

**Figure 7:**
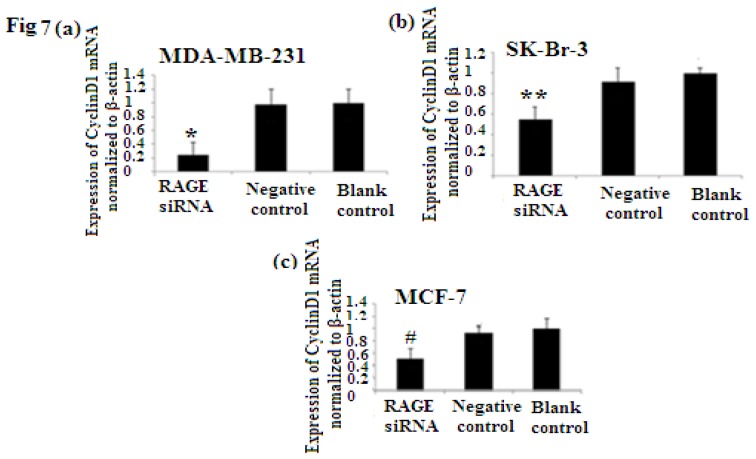

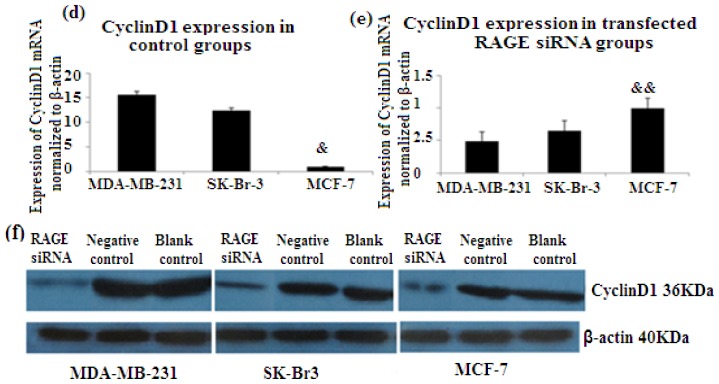
There was decreased expression of CyclinD1 mRNA after transfection with RAGE siRNA as shown by qRT-PCR in MDA-MB-231 (**a**), SK-Br-3 (**b**) and MCF-7 (**c**) cell lines respectively (*, **, # *p* < 0.05) and Western Blot results (**f**) (*p* < 0.05) compared to the negative or blank control groups. Comparison between the expression of CyclinD1 in the three cell lines in the control groups (**d**) and in the transfected groups (**e**). Three independent measurements were performed and averaged.

PCNA (proliferation cell nuclear antigen) is a 36-kilodalton nuclear protein synthesized in the G1/S–phase of the cell cycle and functions as a cofactor for DNA polymerase delta, which is associated with DNA synthesis and repair [59,60]. It is involved in cell cycle regulation and check point control [54]. CyclinD1 is a protein involved in normal cell cycle regulation and is responsible for the transition from the G1 (resting) phase to the S (DNA synthesis) phase. Both PCNA and cyclinD1 are important for the regulation of cell division. qRT-PCR and Western Blot results indicated that RAGE siRNA significantly decreased the expression of PCNA and cyclinD1 mRNA in MCF-7, SK-Br-3 and MDA-MB-231 cells (*p* < 0.5) (Figures 6 and 7).

### 2.7. RAGE siRNA Decreases Expression of NF-κB p65

**Figure 8:**
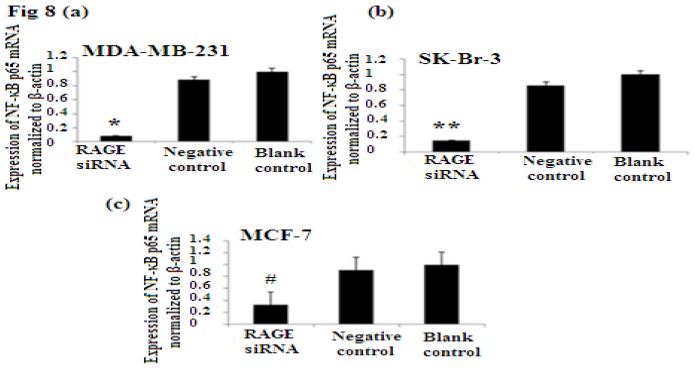

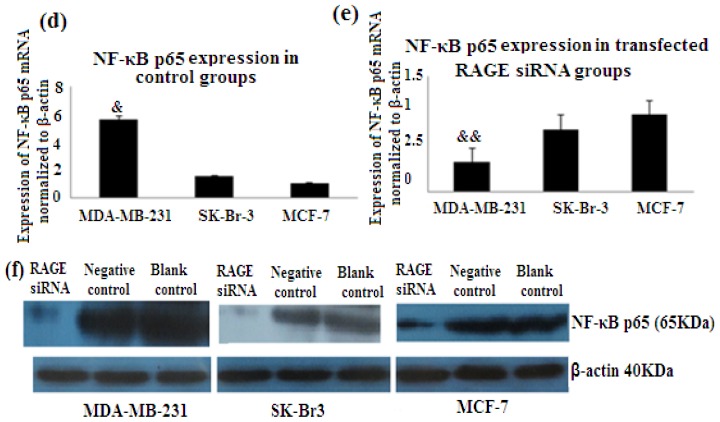
There was decreased expression of NF-κB p65 mRNA after transfection with RAGE siRNA as shown by qRT-PCR in MDA-MB-231 (**a**), SK-Br-3 (**b**) and MCF-7 (**c**) cell lines respectively (*, **, # *p* < 0.05) and Western Blot results (**f**) (*p* < 0.05) compared to the negative or blank control groups. Comparison between the expression of NF-κB p65 in the three cell lines in the control groups (**d**) and in the transfected groups (**e**). Three independent measurements were performed and averaged.

NF-κB is nuclear transcription factor. It regulates cell behavior in many ways. It increases proliferation, inhibits apoptosis, increases inflammatory and immune response [23]. Activation of NF-κB plays role in the development of cancer. In our study we investigated the role of RAGE siRNA on the expression of NF-κB p65. qRT-PCR and Western Blot results demonstrated that RAGE siRNA significantly decreased the expression of NF-κB p65, in MCF-7, SK-Br-3 and MDA-MB-231 cells (*p* < 0.5) (Figure 8).

## 3. Discussion

Breast cancer is considered the most common malignancy affecting women in the world, accounting for 16% of all female cancers (WHO Global Burden of Disease, 2004), and it is the second leading cause of death [1].

RAGE is overexpressed and plays an important role in many pathological conditions [41,53] including diabetes, neuronal degeneration, inflammation, and Alzheimer’s disease [13,28–30]. RAGE is also overexpressed in many types of cancers such as colon cancer [45,46], common bile duct cancer [47], pancreatic cancer [48], prostate cancer [35], and ovarian and brain cancers, as well as lymphoma and melanoma [42]. The interaction between RAGE and its ligands plays a characteristic role in the modulation of cancer cell functions such as increasing tumor invasion and metastasis [3]. RAGE utilizes two pathways during carcinogenesis: the transformation of normal cells into malignant cells and creation of a pro-tumorigenic microenvironment [54]; targeting RAGE is considered a novel strategy for clinical intervention. Despite these findings, few data are available regarding RAGE expression in different breast cancer sub-types and its correlation to breast cancer progression.

MDA-MB-231 (triple-negative basal sub-type) shows remarkably high proliferation [10–12] and has more aggressive clinical behavior with a high recurrence rate than other breast cancer subtypes [13–15,55]. Chemotherapy is the preferred treatment, as it does not respond to trastuzumab or endocrine therapy. Patients with this sub-type also have worse outcomes after chemotherapy than patients with other sub-types [17–19]. Thus, it has been a great challenge to find another line of treatment for this sub-type. In this study, we found that MDA-MB-231 expresses the highest level of RAGE compared with SK-Br-3 or MCF-7. SK-Br-3, which shows poor differentiation [2,21] and has a worse prognosis than luminal breast cancer [2], exhibits moderate expression of RAGE. However, MCF-7 (luminal sub-type), which is less aggressive, showed the lowest levels of RAGE expression. These results suggest that RAGE may have an essential role in the development and malignant proliferation of breast cancer, especially in the triple-negative basal sub-type, which shows more aggressive clinical behavior than other sub-types.

In this study, we demonstrated for the first time that knockdown of RAGE in different sub-types of breast cancer cell lines has a profound effect on proliferation, as shown by MTT assay, and the effect was more significant after 48 h of transfection with RAGE siRNA. This was supported by a significant increase in the number of cells in the G0/G1 phase combined with a decrease in the number of cells in the S and G2 phases. Cell cycle arrest in the G1 phase in MDA-MB-231 and SK-Br-3 (the high and moderate RAGE expressers, respectively) required only a low concentration of siRNA compared to MCF-7 (the low RAGE expresser), which required a higher concentration of siRNA to achieve the significant result. On the other hand, there was significant elevation of biosynthesis and percentage of cell proliferation of the blank controls cells (at S + G2 phases) in the MDA-MB-231 (45%) compared to MCF-7 (29%) and SK-Br-3 (29%). This confirms that the effect of RAGE siRNA on the high RAGE expressing cell line, MDA-MB-231 (which also shows high proliferation), is greater than its effect on the low RAGE expressing cell line, MCF-7 (which also shows low proliferation). If suppression of RAGE by siRNA is feasible and efficient in mouse tumor models, it may facilitate the development of RNA-interfering based therapies. This type of therapy could be more effective if adjuvant with chemotherapy or radiotherapy, which affects cells in cell cycle G0 to G1 [61].

PCNA (proliferation cell nuclear antigen) is a nuclear protein synthesized in the G1/S–phase of the cell cycle and is associated with DNA synthesis and repair [19,59]. Cyclin D1 is a protein involved in normal cell cycle regulation and is responsible for the transition from the G1 (resting) phase to the S (DNA synthesis) phase. Its overexpression promotes transformation to the malignant phenotype. Investigating the expression of both cell regulatory proteins (cyclin D1 and PCNA) demonstrated that there were significant decreases in both PCNA and cyclin D1 expression as a result of RAGE siRNA knockdown in all of the investigated breast cancer cell lines. This further correlates RAGE to cancer cell proliferation and indicates that cell signaling initiated by RAGE leads to higher expression of both cyclinD1 and PCNA.

NF-κB plays significant roles in tumor proliferation [60] and experimental studies verified that NF-κB binding to the cyclin D1 promoter is important for the cyclin D1 regulation [23,61]. In our study, we decreased the expression of RAGE through RAGE targeting siRNA and found a decrease in NF-κB p65 expression which eventually leads to less expression of cyclin D1 and PCNA in all cell types. This also explains why RAGE siRNA arrest cells in G1 phase and decrease DNA synthesis.

Our results showed that the more aggressive cell type and highly proliferated basal sub-type MDA-MB-231 expresses the highest levels of RAGE, NF-κB p65, cyclin D1 and PCNA. This cell sub-type was highly affected by the introduction of RAGE siRNA compared to the other sub-types [Figures 6, 7, 8(d,e)]. This result confirms the role of RAGE as an inducer for cell proliferation and shows that cell signaling started by RAGE leads to higher cell division and ultimately promotion of tumor growth.

Finally, we conclude that there is a reduction in the proliferation of all sub-types of breast cancer that results from RAGE siRNA. This finding is especially pronounced in the aggressive sub-type (basal sub-type, triple-negative) breast cancer. This result correlates RAGE to breast cancer growth and proliferation. Accordingly, interfering with RAGE or RAGE ligands could be helpful to minimize breast cancer proliferation. These data suggest that RAGE represents a novel strategy for the genetic therapeutic intervention of breast cancer, and further study of RAGE siRNA in combination with chemotherapy or radiotherapy is warranted.

## 4. Experimental Section

### 4.1. Cell Culture

The human breast cancer cell line MCF-7 (hormonally dependent breast cancer cell line) was purchased from the Xiangya School of Medicine Cancer Institute; SK-Br-3(HER2 overexpression) was purchased from the Chinese Academy of Sciences, Shanghai Institutes for Biological Sciences Cell Resource Center; and MDA-MB-231(triple-negative) was purchased from Xiangya School of Medicine Cells Center. MCF-7 cells were cultured in Dulbecco’s modified essential medium (DMEM) (Gibco, Carlsbad, CA, USA) while SK-Br-3 and MDA-MB-231 cells were cultured in RPMI 1640 medium (Invitrogen, Carlsbad, CA, USA). Both media were supplemented with 10% heat inactivated fetal bovine serum (Invitrogen, Carlsbad, CA, USA), 100 units/mL ampicillin and 100 mg/mL streptomycin. The cells were incubated at 37 °C and 5% CO_2_.

### 4.2. siRNA Transfection

siRNA were synthesized by Biomics Biotechnologies Company (Shanghai, China). The sequences of the siRNAs were as follows: 5′-GACCAACUCUCUCCUGUAUTT-3′ and 5′-AUACAGGAGAGAGUUGGUCTT-3′. The sequences of negative control RNA were as follows: 5′-UUCUCCGAACGUGUCACGUTT-3′ and 5′-ACGUGACACGUUCGGAGAATT-3′. MCF-7, SK-Br-3, and MDA-MB-231 cells were cultured in 6-well plates until 50%–60% confluence. Cells were transfected with siRNA or the negative control RNA (NC) as follows: 10 μL (3.2 μg) of the siRNA was added into 175 μL of Opti-MEM Reduced-Serum Medium (Invitrogen, Carlsbad, CA, USA) and mixed gently. At the same time, 4 μL of oligofectamin (Invitrogen, Carlsbad, CA, USA) was added into 11 μL of Opti-MEM. After 5 min the Opti-MEM including the siRNA was mixed gently with the Opti-MEM including the oligofectamin. After 20 min, the mixture was added into the wells, and the plates were incubated at 37 °C and 5% CO_2_ for 24 h.

### 4.3. Quantitative Real Time PCR

MCF-7, MDA-MB-231, and SK-Br-3 cells were cultured in 6-well plates, and each cell line was divided into three groups: the siRNA group (transfected with RAGE targeted-siRNA), the negative control group (transfected with negative control RNA to prevent induction of nonspecific cellular events caused by introduction of the oligonucleotide into cells) and the blank control group. After 24 h, culture media were removed, and the cells were washed with PBS. Total RNA purification from cell cultures was performed with the RNeasy Mini Kit (QIAGEN) and converted to cDNA by reverse transcription. qRT-PCR amplifications were performed with a PCR Mastercycler^®^ epgradient Eppendorf (Hamburg, Germany). Amplification was carried out in 20 μL reactions containing 0.8 μL of primer, 10 μL of Platinum SYBR Green qPCR SuperMix-UDG (Invitrogen, Carlsbad, CA, USA), and 1 μL of cDNA. To compare two groups, the ΔCt of each group was calculated by formula ΔCt1 = Ct group1 − Ct β-actin and ΔCt2 = Ct group 2 − Ct β-actin. ΔΔCt was calculated by (ΔCt2 − ΔCt1). The fold-change for gene expression levels was calculated using 2^ΔΔCt^.

The primer sequences used for RAGE qRT-PCR were 5′-GGCTGGTGTTCCCAATAAGG-3′ and 3′-TCACAGGTCAGGGTTACGGTTC-5′;

The primer sequences used for CyclinD1 qRT-PCR were 5′-GACCCCGCACGATTTCAT-3′ and 3′-GGAGGCAGTCTGGGTCACAC-5′;

The primer sequences used for PCNA qRT-PCR were 5′-CAAGAAGGTGTTGGAGGCAC-3′ and 3′-TACTATCGCCAAGGTATCCG-5′;

The primer sequences used for NF-κB P65 qRT-PCR were: 5′-TGCTGTGCGGCTCTGCTTCC-3′ and 3′-AGGCTC GGGTCTGCGTAGGG-5′;

The house keeping gene β-actin was chosen as an endogenous standard to control RNA integrity with the following primers sequences: 5′-GACAGGATGCAGAAGGAGATTACT-3′ and 3′-TGATCCACATCTGCTGGAAGGT-5′.

### 4.4. Western Blot

Western Blot was performed according to the procedure described by Al-Madhagi *et al.*[23]. Briefly, three cell lines, MCF-7, SK-Br-3, and MDA-MB-231, were divided into three groups: siRNA group, the negative control group and blank control group. They were cultured and transfected with RAGE siRNA as described above. After 48 h of incubation, the cell media were removed, and the cells were washed with PBS. Then, the total proteins were extracted from cell cultures using RIPA buffer (Beytime, Shanghai, China). The protein amounts were measured using the BCA method (Beytime, Shanghai, China). Forty micrograms of total protein was loaded and separated on a 12% Acrylamide/Bis gel (Beytime, Shanghai, China), and the protein was transferred to a PVDF membrane. The PVDF membrane was blocked with 5% Bovine Serum Albumin (BSA) in TBST. RAGE mouse monoclonal antibody (Santa-Cruz, California, USA) was diluted in BSA/TBST solution (1:500) and used to detect RAGE protein in cell lysates. The PVDF membrane was incubated with the antibody for 30 min at room temperature and overnight at 4 °C and then washed three times (10 min each) with TBST. Next, the PVDF membrane was incubated with HRP-labeled mouse IgG secondary antibody (Santa-Cruz, CA, USA) diluted in TBST (1:1000) for 1 h and then washed three times (10 min each) with TBST. The protein bands were detected using ECL chemiliumenescent reagents (Beytime, Shanghai, China). Band intensities were measured, and protein signals were normalized with β-actin.

### 4.5. Cell Viability Assay (MTT)

The cell proliferation assay was performed with 3-(4,5-Dimethylthiazol-2-yl)-2, 5-Diphenyltetrazolium Bromide (MTT). MCF-7, SK-Br-3, and MDA-MB-231 cells cultured in 96-well culture plates were transfected with the siRNA as described previously. The cells were then incubated at 37 °C and 5% CO_2_ for 0, 24, 48, and 72 h. At the end of each incubation interval, the cells were washed with PBS buffer, and 10 μL of (5 mg/mL) MTT solution (MTT Cell Proliferation and Cytotoxicity Assay Kit, Beytime, Shanghai, China) was added into each well. The plates were incubated for 4 h at 37 °C and 5% CO_2_. Then, the MTT solution was removed and 150 μL of Formazan was added into each well for 20–30 min. Finally, the absorbance (A value) was measured using a micro-culture plate reader at 490 nm. The cell proliferation inhibition rate by RAGE siRNA was calculated according to the following equation: inhibition rate = [(absorbance value of group1 at 490 nm) − (absorbance value of group 2 at 490 nm)/absorbance value of group 1 at 490 nm] × 100. Each assay was performed in triplicate, and the average absorbance was calculated.

### 4.6. Cell Cycle Analysis

MCF-7, SK-Br-3, and MDA-MB-231 cells cultured in 96-well culture plates were transfected with different concentrations (3.2 μg, 6.4 μg or 9.6 μg) of siRNA as described above. The cells were then incubated at 37 °C and 5% CO_2_ for 48 h. Transfected cells were harvested with trypsin, washed with 4 °C PBS one time, and fixed with 75% cold ethanol at 4 °C. The cells were washed with PBS again and centrifuged. After that the cells were re-suspended with PBS, 0.5 mg/mL RNase A solution (Beytime, Shanghai, China) was added for 1 h at 37 °C, and the cells were stained with 10 μg/mL propidium iodide (Beytime, Shanghai, China) for 30 min at 4 °C in dark. The stained cells were analyzed by BD-FACS caliber flow cytometry (BD Biosciences, San Jose, CA, USA). The proportion of cells in each phase of the cell cycle was determined by a FACS scan for Quantitative Cell Analysis.

### 4.7. Apoptosis Assay

MCF-7, SK-Br-3, and MDA-MB-231 cells were cultured in 6-well culture plates and transfected with siRNA as described above. The cells were then incubated at 37 °C and 5% CO_2_ for 48 h. Transfected cells were harvested with trypsin, washed twice with cold PBS and then re-suspended in binding buffer. After that, cells were stained with Annexin V-FITC and propidium iodide using the Annexin V-FITC Apoptosis Detection Kit I. FACS Calibur Flow cytometer was used to quantify the percentage of apoptotic cells.

### 4.8. Statistical Analysis

Statistical analyses were carried out using SPSS 11.5 software package (SPSS Inc., Chicago, IL, USA) for Windows and are presented as the means ± SE. One-way ANOVA was used to compare the data among three or more groups. Student’s *t*-test was used to compare two groups. In all analyses, a *p* value less than 0.05 was considered statistically significant. Image J software (NIH, Bethesda, MD, USA) was used to measure the size and percentage of protein bands on the films for Western Blotting.

## 5. Conclusions

In conclusion, transfection with RAGE siRNA leads to significant reduction in the proliferation of all sub-types of breast cancer, especially in the aggressive sub-type (triple-negative). Breast cancer growth and proliferation are correlated to RAGE expression levels. Thus, RAGE knockdown should be considered as a novel treatment option to enhance therapeutic efficacy in breast cancer. Further studies of RAGE in combination with chemotherapy or radiotherapy are needed.
